# Oxidative phosphorylation and lacunar stroke: Genome-wide enrichment analysis of common variants

**DOI:** 10.1212/WNL.0000000000002260

**Published:** 2016-01-12

**Authors:** Matthew Traylor, Christopher D. Anderson, Robert Hurford, Steve Bevan, Hugh S. Markus

**Affiliations:** From Clinical Neurosciences (M.T., R.H., H.S.M.), University of Cambridge, UK; School of Life Science (S.B.), University of Lincoln, UK; and the Center for Human Genetic Research (C.D.A.), Department of Neurology, Massachusetts General Hospital, Boston.

## Abstract

**Objective:**

We investigated whether oxidative phosphorylation (OXPHOS) abnormalities were associated with lacunar stroke, hypothesizing that these would be more strongly associated in patients with multiple lacunar infarcts and leukoaraiosis (LA).

**Methods:**

In 1,012 MRI-confirmed lacunar stroke cases and 964 age-matched controls recruited from general practice surgeries, we investigated associations between common genetic variants within the OXPHOS pathway and lacunar stroke using a permutation-based enrichment approach. Cases were phenotyped using MRI into those with multiple infarcts or LA (MLI/LA) and those with isolated lacunar infarcts (ILI) based on the number of subcortical infarcts and degree of LA, using the Fazekas grading. Using gene-level association statistics, we tested for enrichment of genes in the OXPHOS pathway with all lacunar stroke and the 2 subtypes.

**Results:**

There was a specific association with strong evidence of enrichment in the top 1% of genes in the MLI/LA (subtype *p* = 0.0017) but not in the ILI subtype (*p* = 1). Genes in the top percentile for the all lacunar stroke analysis were not significantly enriched (*p* = 0.07).

**Conclusions:**

Our results implicate the OXPHOS pathway in the pathogenesis of lacunar stroke, and show the association is specific to patients with the MLI/LA subtype. They show that MRI-based subtyping of lacunar stroke can provide insights into disease pathophysiology, and imply that different radiologic subtypes of lacunar stroke subtypes have distinct underlying pathophysiologic processes.

Accumulating evidence suggests that changes in mitochondrial function influence risk of stroke, particularly for the ischemic and hemorrhagic stroke subtypes arising from cerebral small vessel disease.^1–3^ The mitochondrial genome is essential for the assembly of the oxidative phosphorylation (OXPHOS) apparatus, consisting of 5 protein complexes necessary for maintenance of aerobic haemostasis.^4^ Genetic variants lying within genes encoding the OXPHOS apparatus, the majority of which reside in the autosome, have been associated with risk of lacunar stroke and deep intracerebral hemorrhage.^1–3^ Additionally, multiple rare disorders are caused by mutations in OXPHOS genes,^5^ many of which result in stroke-like episodes, neurodegeneration, and leukoencephalopathies.

Neuropathologic studies suggest that lacunar stroke results from a number of differing vascular pathologies, including focal atherosclerosis, often associated with isolated larger lacunar infarcts, and a more diffuse arteriopathy, usually seen in hypertensive individuals.^6^ The diffuse arteriopathy has been associated both pathologically and neuroradiologically with multiple lacunar infarcts, as well as confluent leukoaraiosis (LA) on MRI.^7^ Given the considerations described above, one might hypothesize that genetic variation within OXPHOS is more likely to be associated with patients with LA or multiple infarcts.

We investigated whether common genetic variants within the OXPHOS pathway were involved in the pathogenesis of MRI-confirmed lacunar stroke using a permutation-based enrichment approach, evaluating the strength of genetic associations within genes in the OXPHOS complex compared to the background of random genes across the autosome, and determining whether strength of any associations differed by lacunar stroke subtype.

## METHODS

### Study population.

A total of 1,029 Caucasian patients with lacunar stroke, aged ≤70 years, were recruited from 72 specialist stroke centers throughout the United Kingdom (supplementary material on the *Neurology®* Web site at Neurology.org), between 2002 and 2012, as part of the Young Lacunar Stroke DNA Resource. Lacunar stroke was defined as a clinical lacunar syndrome,^8^ with an anatomically compatible lesion on MRI seen as either acute on diffusion-weighted imaging or low signal on T1 or fluid-attenuated inversion recovery imaging and with diameter ≤15 mm. All patients underwent full stroke investigation, including brain MRI, imaging of the extracerebral arteries, arteries, and ECG. Echocardiography was performed when appropriate. All MRIs and clinical histories were reviewed centrally by one physician (H.S.M.). Exclusion criteria were as follows: stenosis >50% in the extracranial or intracranial cerebral vessels or previous carotid endarterectomy; cardioembolic source of stroke, defined according to the Trial of Org 10172 in Acute Stroke Treatment criteria^9^ as high or moderate probability; cortical infarct on MRI; subcortical infarct >15 mm in diameter, as these can be caused by embolic mechanisms (striatocapsular infarcts); and any other specific cause of stroke (e.g., lupus anticoagulant, cerebral vasculitis, dissection, monogenic cause of stroke). All cases were screened for *NOTCH3* cerebral autosomal dominant arteriopathy with subcortical infarcts and leukoencephalopathy and Fabry disease mutations and positive cases were excluded. An additional 82 Caucasian patients with lacunar stroke were recruited from St. George's Hospital, London, as part of the GENESIS study. The same inclusion and exclusion criteria were used as in the DNA-lacunar study except that older patients were also included, and a similar investigation protocol was used with all patients having brain MRI.

Unrelated Caucasian controls, free of clinical cerebrovascular disease, were obtained by random sampling from general practice lists from 4 sites in England and Scotland and chosen to match the same geographical location as the patients. Sampling was stratified for age and sex. All patients and controls underwent a standardized clinical assessment and completed a standardized study questionnaire with the same risk factor definitions as used for the cases. MRI was not performed in controls.

### Standard protocol approvals, registrations, and patient consents.

The study was approved by the Multi-Centre Research Ethics Committee (04/MRE00/36) and informed consent was obtained from all participants.

### Subtyping of lacunar stroke.

We used the Fazekas scale, a semiquantitative rating scale that classifies LA into 4 groups ranging from none (0) to severe (3), to grade the extent of LA on MRI. Based on this grading, we separated the patients into 2 strata: (1) isolated lacunar infarct (ILI): patients with only a single infarct and mild or absent LA (Fazekas grade <2); (2) multiple lacunar infarcts (MLI)/LA: MLI or moderate to severe LA (Fazekas grade ≥2). We randomly selected 20 MRI scans to be graded for a second time by the same rater. Perfect agreement was found in assignment to either of the 2 groups (κ = 1). In addition, to evaluate inter-rater reliability, we randomly selected 40 scans, which were graded by a second rater (R.H.). Perfect agreement was found between the 2 raters for allocation of lacunar subtype (κ = 1). However, one scan was graded MLI by one rater and LA by the other.

### Genotyping and imputation.

All included samples were genotyped on the Illumina (San Diego, CA) HumanExomeCore array. Single nucleotide polymorphisms (SNPs) were excluded if they had minor allele frequency <0.01, had genotype missingness >3%, had Hardy-Weinberg equilibrium *p* < 1e-6 in controls, were strand ambiguous (A/T or C/G), or showed evidence of differential missingness by case-control status (*p* < 0.05). Individuals were excluded if they had missingness >3%, had excess or reduced heterozygosity, showed evidence of relatedness with another individual (pi-hat > 0.1875), or failed a sex check in PLINK. To assess population ancestry, all individuals were merged with the HapMap II populations (CEU, YRI, JPT + CHB). The merged dataset was linkage disequilibrium (LD) pruned and regions with long-range LD were removed. Ancestry-informative principal components were determined using EIGENSTRAT's smartpca function.^10^ A total of 284 individuals were removed who did not segregate with CEU HapMap II individuals. We then repeated smartpca on the remaining Caucasian individuals. We removed all individuals who were more than 6 standard deviations from the mean on the first 2 principal components of the first 8 iterations of smartpca. The remaining 269,691 autosomal SNPs and 2,603 individuals were then imputed to 1000 Genomes phase 1 (March 2012)^11^: SHAPEIT v2 was used to phase the haplotypes and IMPUTE v2.2.2 was used to perform the imputation,^12,13^ resulting in 9,289,526 SNPs.

### Enrichment analysis.

We first tested genome-wide association with each autosomal SNP, including the first 2 principal components as covariates, using SNPTEST v.2.4. From the genome-wide association statistics, we calculated gene-level statistics for each autosomal gene using the VEGAS software package.^14^ VEGAS simulates SNP associations based on the LD structure of each gene (±50 Kb window) and calculates an empirical *p* value for association of each gene with disease status based on the proportion of simulations in which the sum of simulated SNP χ^2^ statistics exceeds the observed sum from the association results.

**Figure 1:**
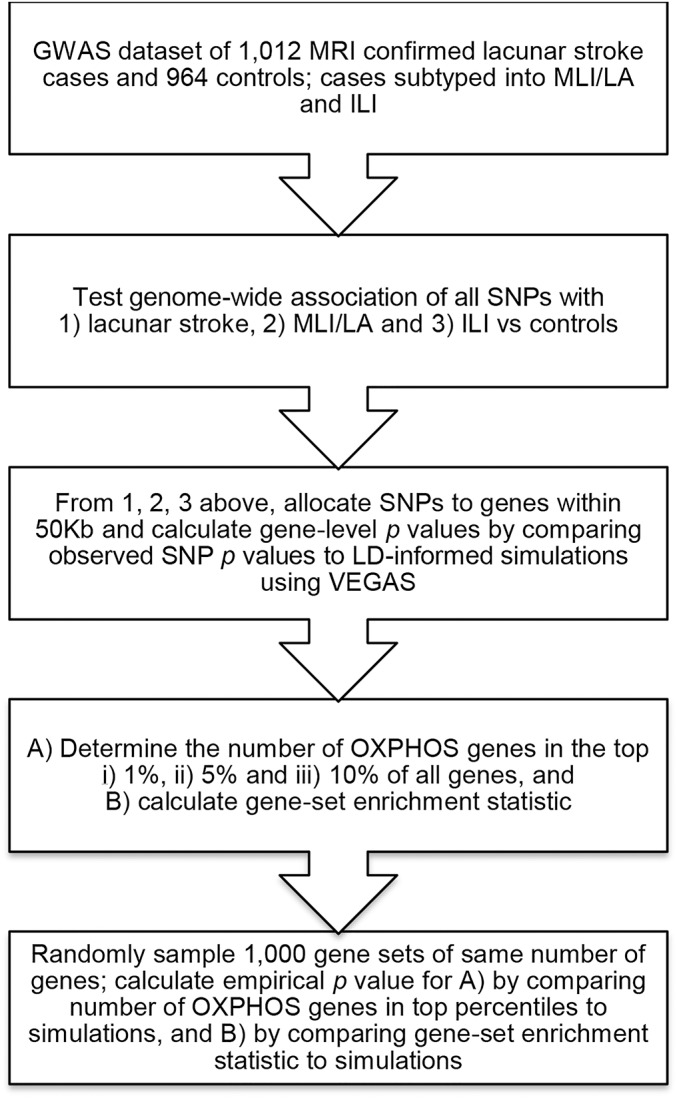
Flowchart of analyses performed GWAS = genome-wide association study; ILI = isolated lacunar infarct; LA = leukoaraiosis; LD = linkage disequilibrium; MLI = multiple lacunar infarcts; OXPHOS = oxidative phosphorylation pathway; SNP = single nucleotide polymorphism.

We then used the generated gene-level statistics to test for significant enrichment of the OXPHOS genes compared to random sets of genes of the same number. We obtained the gene-level results (from VEGAS) for all OXPHOS genes, and tabulated the number of genes from the pathway in the top 1%, 5%, and 10% of all genes. We then generated 1,000 gene sets of the same length, sampling randomly from all gene level results from VEGAS (increased to 10,000 permutations for *p* < 0.05). We calculated significance by determining the proportion of randomly permuted gene sets in which as many or more genes were from the given percentile of all genes. In addition, we used the gene-set enrichment analysis algorithm (GSEA) to test for overall enrichment across all genes, simulating as previously.^15^ We first performed all analyses for all lacunar stroke cases vs controls, and then for the 2 subtypes of ILI or MLI/LA vs controls (figure 1). We set a significance threshold of *p* = 0.0042, corresponding to Bonferroni correction for the 12 analyses (3 phenotypes, 4 tests).

## RESULTS

Details of the study population are given in table 1; 1,012 cases and 964 controls remained after genotyping quality control steps, with 502 cases in the MLI/LA subtypes and 501 in the ILI subtype; in 9 cases, clear separation into different phenotypes was not possible based on brain imaging. We calculated gene-based statistics for 17,656 autosomal genes in each of the analyses using the VEGAS package.^14^ Using these gene-level association statistics, we tested for enrichment of genes in the OXPHOS pathway with lacunar stroke. We found a near-significant enrichment (*p* = 0.07) of genes in the top percentile with all lacunar stroke analysis (table 2). We then performed secondary analyses in the MLI/LA and ILI subtypes. This showed strong evidence of enrichment in the top 1% of genes with the MLI/LA subtype (*p* = 0.0017). Conversely, we found no association with the ILI subtype (*p* = 1). The OXPHOS genes in the top 1% for the MLI/LA subtyped consisted of *NDUFB2*, *SURF1*, *UQCRH*, *NDUFS1*, and *ATP5I*. Plots of the *p* values in these gene regions by genomic position are provided in figure e-1. We followed up our significant result with MLI/LA by testing for enrichment in any of the OXPHOS subcomplexes (table 2). However, we could find no evidence that enrichment was specific to any subcomplex. Indeed, the 5 OXPHOS genes in the top 1% for MLI/LA were divided between OXPHOS subcomplexes: 2 were in complex I (*NDUFB2*, *NDUFS1*), 1 was in complex II (*UQCRH*), 1 was in complex IV (*SURF1*), and 1 was in complex V (*ATP5I*). We found no significant enrichment in the top 5% or 10% of genes, or when using the GSEA algorithm (table 2).

**Table 1:**
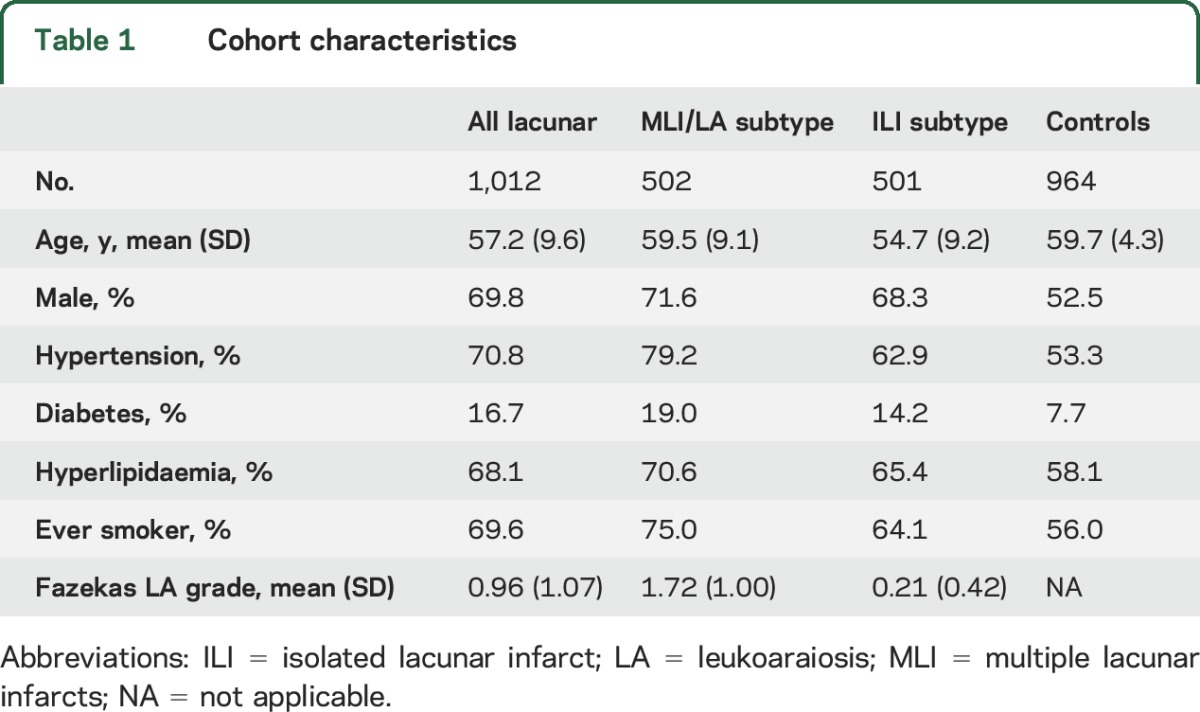
Cohort characteristics

**Table 2:**
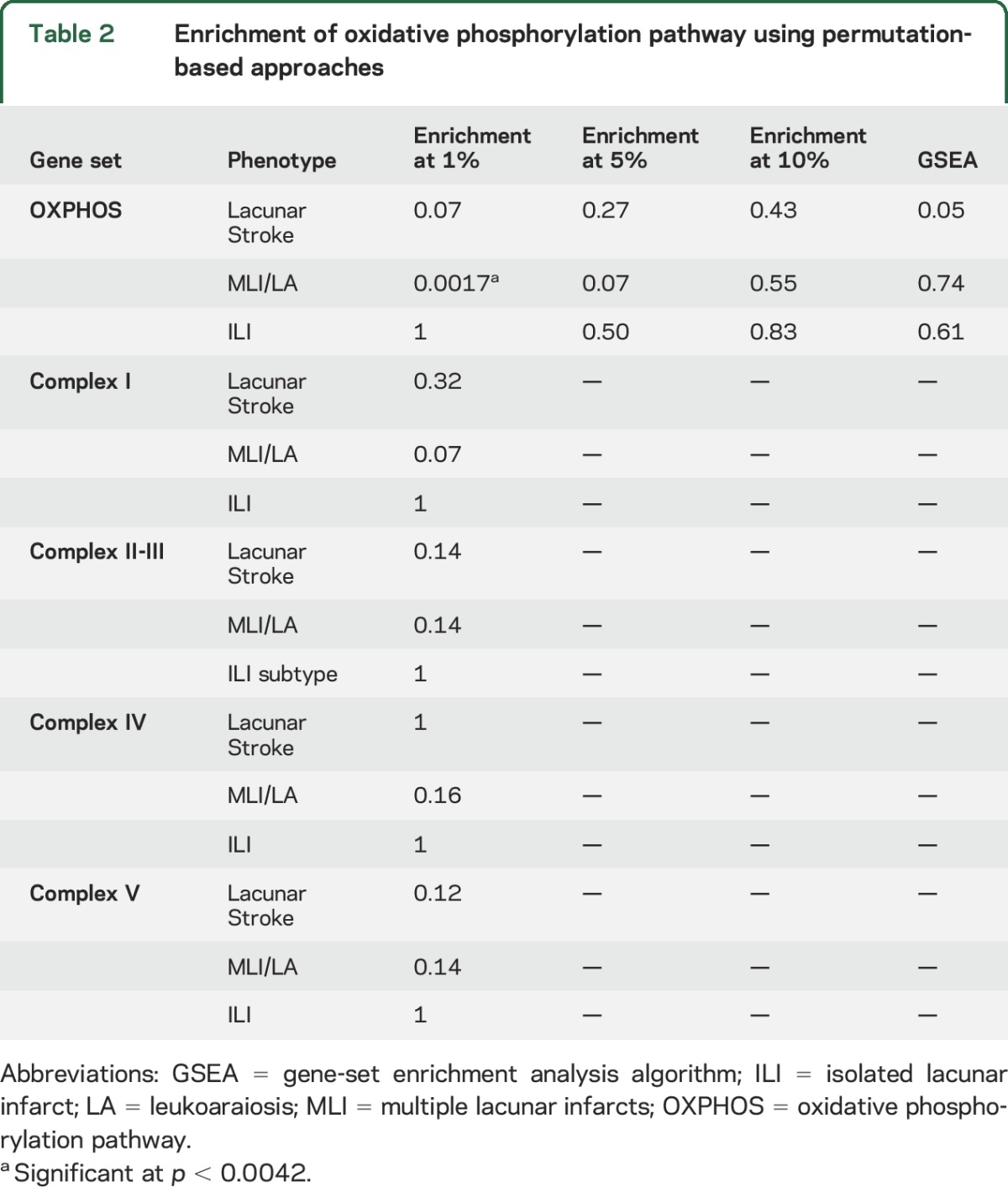
Enrichment of oxidative phosphorylation pathway using permutation-based approaches

## DISCUSSION

In this genome-wide enrichment analysis of MRI-confirmed lacunar stroke cases and age-matched controls, we evaluated an association of the oxidative phosphorylation pathway with lacunar stroke, finding an association in cases with LA or multiple lacunar infarcts, with no evidence for association in cases with isolated lacunar infarcts. The present study extends the previous findings by Anderson et al.,^3^ and defines the phenotypes driving this previously reported genetic association.

Although our approach highlights an aggregate association and therefore cannot identify specific causal genetic variants, these results have important implications. First, they provide evidence for the association of the OXPHOS pathway with lacunar stroke, and importantly show that the association is specific and limited to a subgroup of SVD patients, namely those with multiple lacunar infarcts/LA. Potential mechanisms by which OXPHOS dysfunction increases risk of lacunar stroke are numerous, including depletion of ATP, generation of reactive oxygen species, alterations in autophagy, or changes to cell signaling. Mitochondrial dysfunction and oxidative stress in endothelial cells have been shown to result in increased blood–brain barrier permeability, as well as to increase the expression of adhesion molecules.^16,17^ One intriguing hypothesis is that mitochondrial dysfunction leads to endothelial dysfunction through nitric oxide (NO) dysregulation. A similar effect has been seen in the mitochondrial encephalomyopathy, lactic acidosis, and stroke-like episodes (MELAS), where citrulline and arginine supplementations have shown improved outcomes by ameliorating NO production.^18,19^ Given that white matter disease imaging phenotypes are present in both MELAS and our MLI/LA cohort, this latter mechanism may deserve further scrutiny in functional follow-up studies building on these results.

Second, our results highlight the importance of careful subtyping of stroke cases, demonstrating that detailed phenotyping with MRI can provide valuable insights into disease pathogenesis. In this case, subtyping lacunar strokes using a classification scheme supported by previous histopathologic assessments has allowed the detection of novel associations between OXPHOS and MLI/LA. In addition, our results have implications for pathogenesis of small vessel disease as they imply that pathologic differences between lacunar stroke subtypes are due to distinct underlying pathophysiologic processes. Given that Biffi et al.^20^ further tested shared OXPHOS associations between ischemic stroke and Alzheimer disease, additional studies will be needed to determine whether the associations between OXPHOS genetic variation and MLI/LA stroke can extend to neurodegenerative disease as well, and whether OXPHOS abnormalities are specific to MLI/LA or influence other manifestations of cerebral small vessel disease such as enlarged perivascular spaces and microbleeds.

This study has several strengths. All lacunar stroke cases were MRI confirmed. All individuals, cases and controls, were genotyped on the same genome-wide association study array, which means that the results are less likely to be confounded by technical artifacts. Our study also has limitations. We were unable to obtain MRIs in the control population for this analysis. Population-based studies show that a subset of these are likely to have some degree of small vessel disease. This may have some effect on our results, with our results underestimating the true difference between cases and controls. Second, no adequately sized replication dataset with MRI-verified lacunar stroke is currently available to replicate our findings, so we were unable to independently validate our findings. Similarly, our analysis was limited to individuals of Caucasian ancestry from the United Kingdom. Extension of this analysis to other populations will be an important future development. Future studies will be needed to independently replicate our findings, and to identify specific causal variants and how they lead to OXPHOS dysfunction. Finally, we cannot identify whether specific cell types (i.e., endothelial cells, neurons, glia) are particularly affected by the OXPHOS variants tested in this analysis, which would be helpful in planning functional follow-up studies.

## Supplementary Material

Data Supplement

## GLOSSARY

GSEA: gene-set enrichment analysis algorithm
ILI: isolated lacunar infarct
LA: leukoaraiosis
LD: linkage disequilibrium
MELAS: mitochondrial encephalomyopathy, lactic acidosis, and stroke-like episodes
MLI: multiple lacunar infarcts
NO: nitric oxide
OXPHOS: oxidative phosphorylation
SNP: single nucleotide polymorphism
