# Supplementary material for: Oxidative phosphorylation and lacunar stroke: Genome-wide enrichment analysis of common variants
Source: Neurology. 2016 Jan 12;86(2):141–5. doi: 10.1212/WNL.0000000000002260 (PMC4731691; doi:10.1212/WNL.0000000000002260)
Supplement: Data Supplement [file supp_86_2_141__index.html]

Data Supplement 

# Oxidative phosphorylation and lacunar stroke

## Data Supplement

One figure and supplemental data; one PDF file and one Microsoft Word file.

**Neurology® data supplements are not copyedited before publication. Published editorials and translations have been copyedited.  
 © 2016 American Academy of Neurology.  
  
 Files in this Data Supplement:**

- Figure e-1 - PDF file
- Supplemental data - Microsoft Word file
