# Supplementary material for: Oxidative phosphorylation and lacunar stroke: Genome-wide enrichment analysis of common variants
Source: Neurology. 2016 Jan 12;86(2):141–5. doi: 10.1212/WNL.0000000000002260 (PMC4731691; doi:10.1212/WNL.0000000000002260)
Supplement: Data Supplement [file supp_WNL.0000000000002260_Figure_e-1.pdf]

Figure e-1. Plots of the p-values in gene regions by genomic position

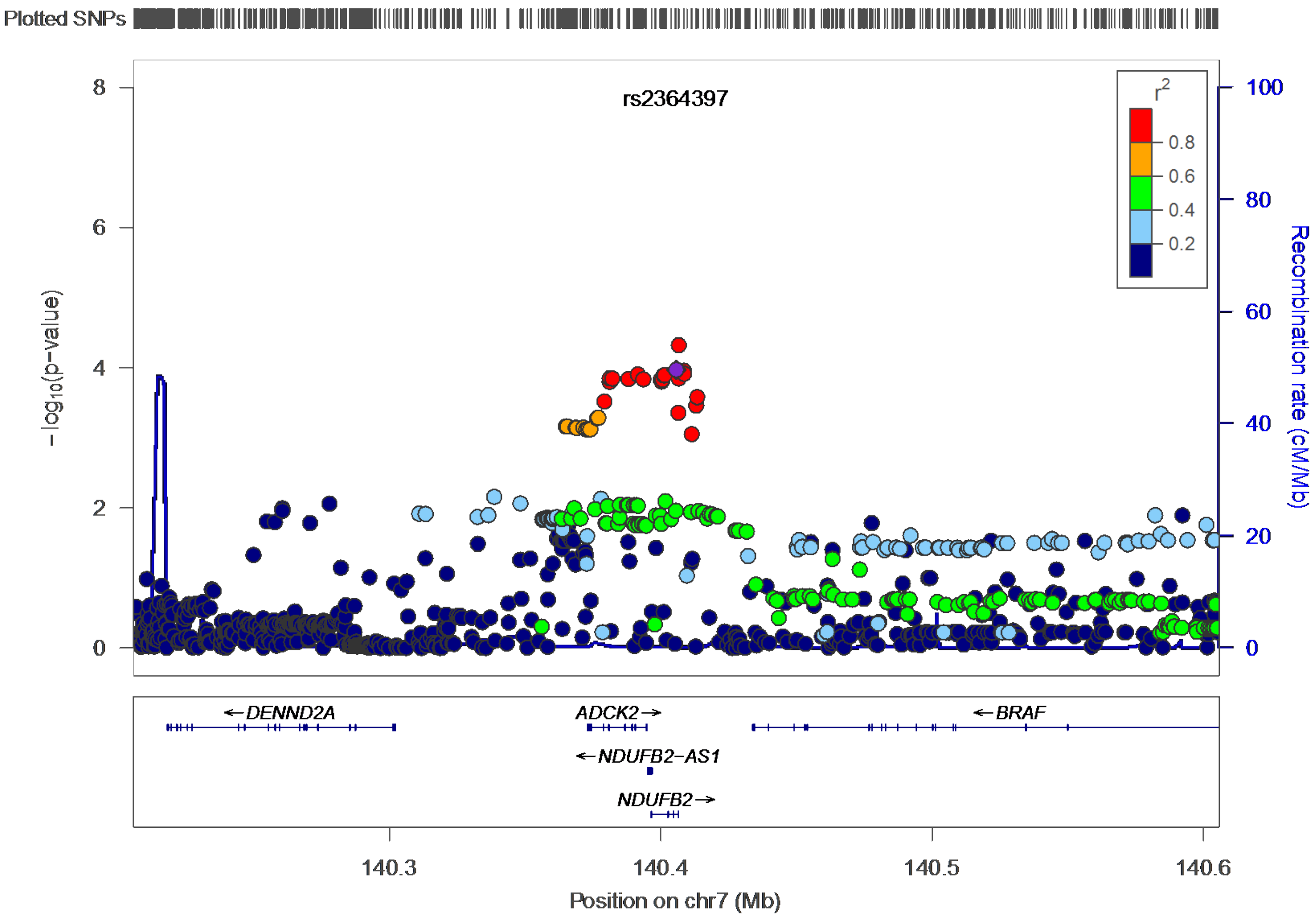

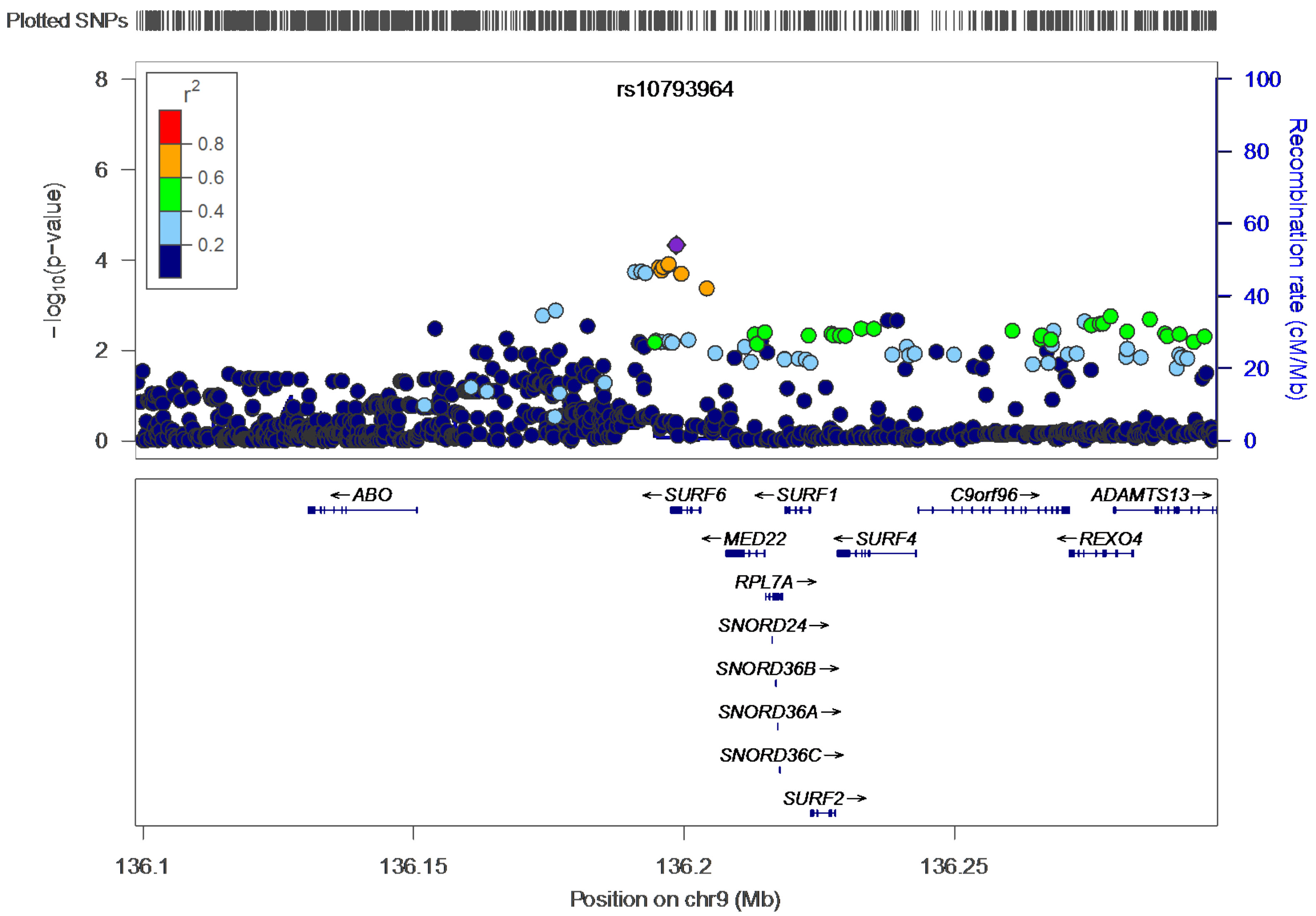
Plotted SNPs
rs10793964
r2
0.8
0.6
0.4
0.2
−log10(p-value)
Recombination rate (cM/Mb)
ABO
SURF6
SURF1
C9orf96
ADAMTS13
MED22
SURF4
REXO4
RPL7A
SNORD24
SNORD36B
SNORD36A
SNORD36C
SURF2
136.1
136.15
136.2
136.25
Position on chr9 (Mb)

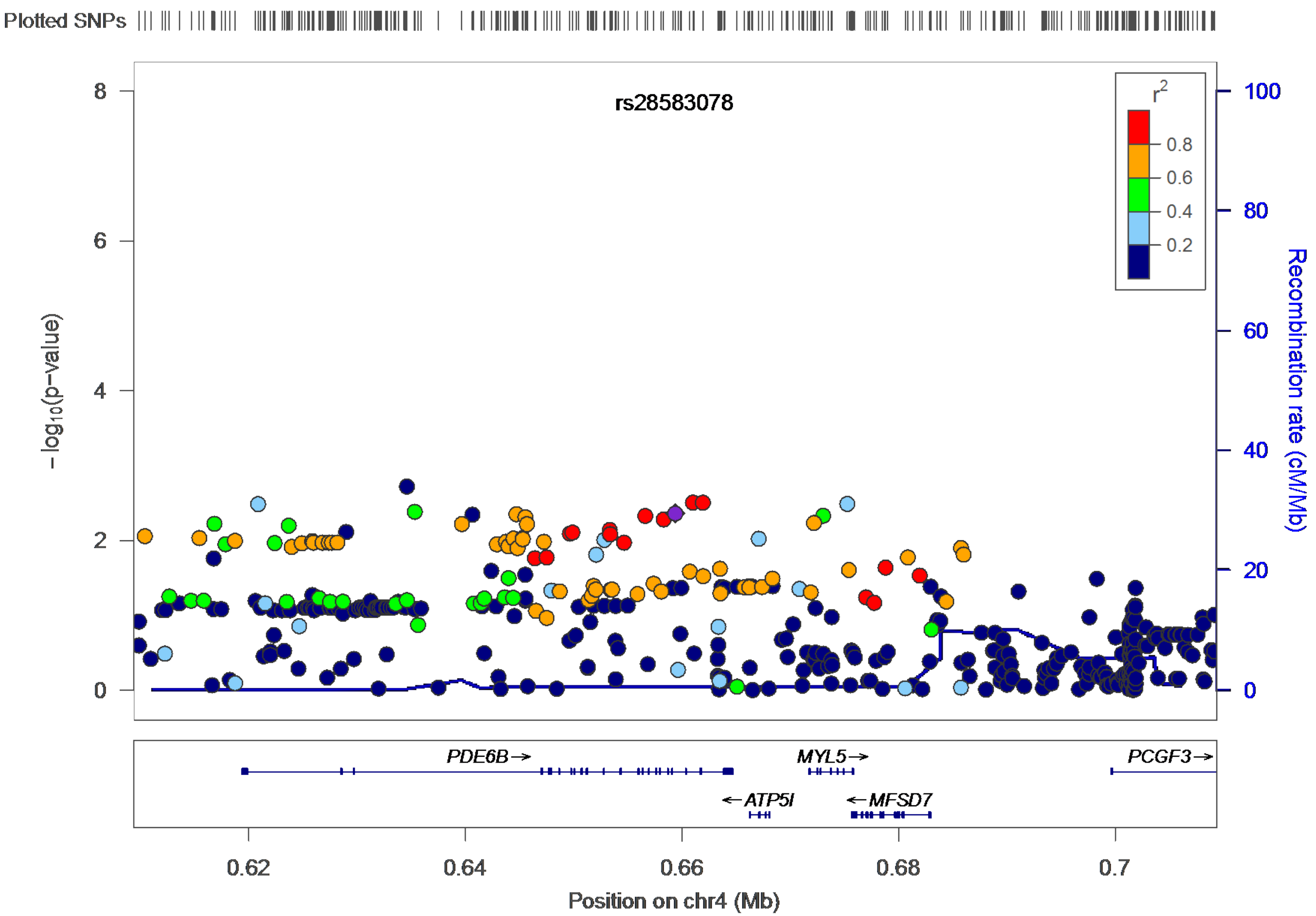
Plotted SNPs
rs28583078
r2
0.8
0.6
0.4
0.2
−log10(p−value)
8
6
4
2
0
Recombination rate (cM/Mb)
100
80
60
40
20
0
PDE6B→
MYL5→
PCGF3→
←ATP5I
←MFSD7
0.62
0.64
0.66
0.68
0.7
Position on chr4 (Mb)

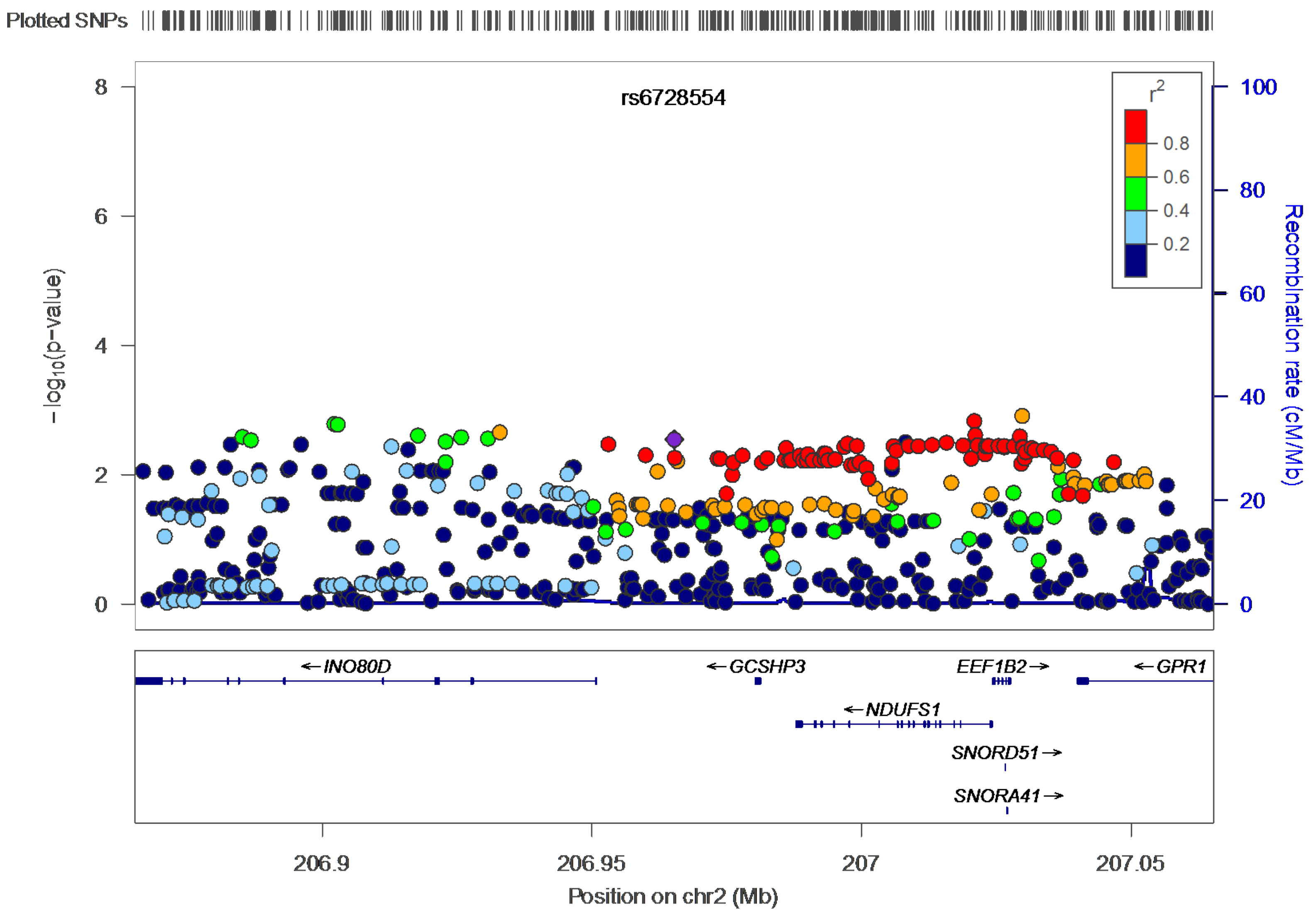

Plotted SNPs
rs6728554
r2
0.8
0.6
0.4
0.2
8
6
4
2
0
100
80
60
40
20
0
-log10(p-value)
Recombination rate (cM/Mb)
INO80D
GCSHP3
EEF1B2
GPR1
NDUFS1
SNORD51
SNORA41
206.9
206.95
207
207.05
Position on chr2 (Mb)

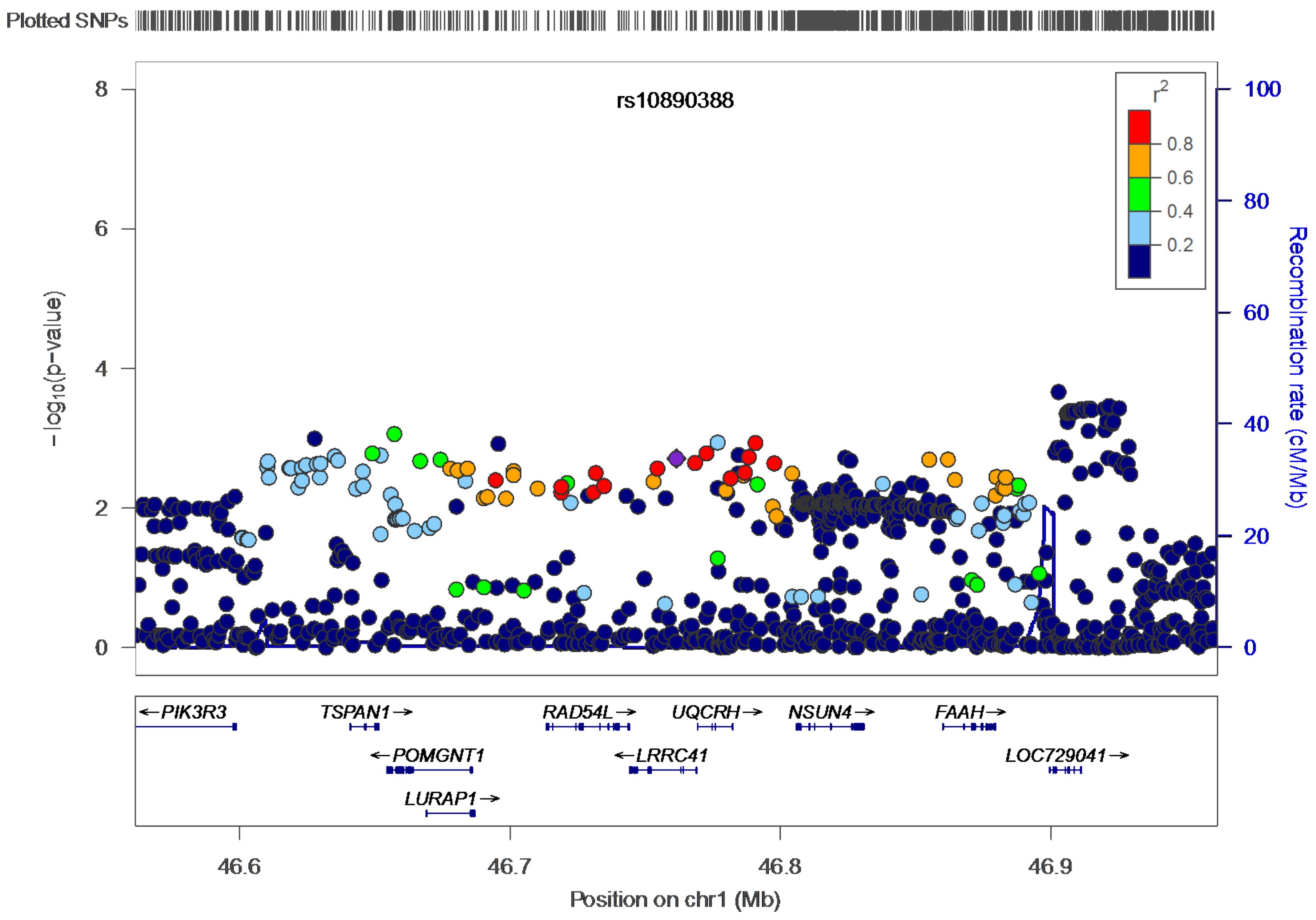

Plotted SNPs
rs10890388
r2
0.8
0.6
0.4
0.2
8
6
4
2
0
−log10(p-value)
100
80
60
40
20
0
Recombination rate (cM/Mb)
←PIK3R3
TSPAN1→
←POMGNT1
LURAP1→
RAD54L→
←LRRC41
UQCRH→
NSUN4→
FAAH→
LOC729041→
46.6
46.7
46.8
46.9
Position on chr1 (Mb)
