# Supplementary material for: Oxidative phosphorylation and lacunar stroke: Genome-wide enrichment analysis of common variants
Source: Neurology. 2016 Jan 12;86(2):141–5. doi: 10.1212/WNL.0000000000002260 (PMC4731691; doi:10.1212/WNL.0000000000002260)
Supplement: Data Supplement [file supp_WNL.0000000000002260_Supplemental_data.docx]

**Acknowledgements**

*Study managers:* Josie Monaghan; Alan Zanich, Samantha Febrey, Eithne Smith, Jenny Lennon, St George’s University of London

*Database cleaning:* Loes Rutten-Jacobs, University of Cambridge

*Participating centres (number of enrolled patients per centre; local investigators)*:

Aberdeen Royal Infirmary, Aberdeen (12; Mary Macleod). Addenbrooke’s Hospital, Cambridge (54; Jean-Claude Baron, Elizabeth Warburton, Diana J Day, Julie White). Airedale General Hospital, Steeton (4; Samantha Mawer). Barnsley Hospital, Barnsley (3; Mohammad Albazzaz, Pravin Torane, Keith Elliott, Kay Hawley). Bart’s and the London, London (2; Patrick Gompertz). Basingstoke and North Hampshire Hospital, Basingstoke (13; Elio Giallombardo, Deborah Dellafera). Blackpool Victoria Hospital, Blackpool (11; Mark O'Donnell). Bradford Royal Infirmary, Bradford (1; Chris Patterson). Bristol Royal Infirmary, Bristol (8; Sarah Caine). Charing Cross Hospital, London (12; Pankaj Sharma). Cheltenham General and Gloucester Royal Hospitals, Cheltenham and Gloucester (10; Dipankar Dutta). Chesterfield Royal Hospital, Chesterfield (4; Sunil Punnoose, Mahmud Sajid). Countess of Chester Hospital, Chester (22; Kausik Chatterjee). Derriford Hospital, Plymouth (4; Azlisham Mohd Nor). Dorset County Hospital NHS Foundation Trust, Dorchester (6; Rob Williams). East Kent Hospitals University NHS Foundation Trust, Kent (22; Hardeep Baht, Guna Gunathilagan). Eastbourne District General Hospital, Eastbourne (4; Conrad Athulathmudali). Frenchay Hospital, Bristol (1; Neil Baldwin). Frimley Park Hospital NHS Foundation Trust, Frimley (6; Brian Clarke). Guy’s and St Thomas’ Hospital, London (14; Tony Rudd). Institute of Neurology, London (25; Martin Brown). James Paget University Hospital, Great Yarmouth (1; Peter Harrison). King's College Hospital, London (16; Lalit Kalra). Leeds Teaching Hospitals NHS Trust, London (125; Ahamad Hassan). Leicester General Hospital and Royal Infirmary, Leicester (9; Tom Robinson, Amit Mistri). Luton and Dunstable NHSFT University Hospital, Luton (16; Lakshmanan Sekaran, Sakthivel Sethuraman, Frances Justin). Maidstone andTunbridge Wells NHS Trust (3; Peter Maskell). Mayday University Hospital, Croydon (14; Enas Lawrence). Medway Maritime Hospital, Gillingham (5; Sam Sanmuganathan). Milton Keynes Hospital, Milton Keynes (1; Yaw Duodu). Musgrove Park Hospital, Taunton (9; Malik Hussain). Newcastle Hospitals NHS Foundation Trust, Newcastle upon Tyne (12; Gary Ford). Ninewells Hospital, Dundee (5; Ronald MacWalter). North Devon District Hospital, Barnstaple (8; Mervyn Dent). Nottingham University Hospitals, Nottingham (17; Philip Bath, Fiona Hammonds). Perth Royal Infirmary, Perth (2; Stuart Johnston). Peterborough City Hospital, Peterborough (1; Peter Owusu-Agyei). Queen Elizabeth Hospital, Gateshead (5; Tim Cassidy, Maria Bokhari). Radcliffe Infirmary, Oxford (5; Peter Rothwell). Rochdale Infirmary, Rochdale (4; Robert Namushi). Rotherham General Hospital, Rotherham (1; James Okwera). Royal Cornwall Hospitals NHS Trust, Truro (11; Frances Harrington, Gillian Courtauld). Royal Devon and Exeter Hospital, Exeter (22; Martin James). Royal Hallamshire Hospital, Sheffield (1; Graham Venables). Royal Liverpool University Hospital and Broadgreen Hospital, Liverpool (9; Aravind Manoj). Royal Preston Hospital, Preston (18; Shuja Punekar). Royal Surrey County Hospital, Guildford (23; Adrian Blight, Kath Pasco). Royal Sussex County Hospital, Brighton (14; Chakravarthi Rajkumar, Joanna Breeds). Royal United Hospital, Bath (6; Louise Shaw, Barbara Madigan). Salford Royal Hospital, Salford (16; Jane Molloy). Southampton General Hospital, Southampton (1; Giles Durward). Southend Hospital, Westcliff-on-Sea (26; Paul Guyler). Southern General Hospital, Glasgow (34; Keith Muir, Wilma Smith). St George’s Hospital, London (108; Hugh Markus). St Helier Hospital, Carshalton (10; Val Jones). Stepping Hill Hospital, Stockport (4; Shivakumar Krishnamoorthy). Sunderland Royal Hospital, Sunderland (1; Nikhil Majumdar). The Royal Bournemouth Hospital, Bournemouth (15; Damian Jenkinson). The Walton Centre, Liverpool (15; Richard White). Torbay Hospital, Torquay (19; Debs Kelly). University Hospital Aintree, Liverpool (19; Ramesh Durairaj). University Hospital of North Staffordshire, Stoke-on-trent (16; David Wilcock). Wansbeck General Hospital and North Tyneside Hospital, Ashington and North Shields (6; Christopher Price). West Cumberland Hospital, Whitehaven (6; Olu Orugun, Rachel Glover). West Hertfordshire Hospital, Watford (20; David Collas). Western General Hospital, Edinburgh (12; Cathie Sudlow). Western Infirmary, Glasgow (33; Kennedy R. Lees, Jesse Dawson). Wycombe Hospital and Stoke Mandeville, High Wycombe (20; Dennis Briley and Matthew Burn). Yeovil District Hospital, Yeovil (46; Khalid Rashed). York Teaching Hospital, York (1; John Coyle).
